# Preferences regarding preconceptional and genetic counselling and pregnancy check-ups among women with the *PLN* p. (Arg14del) variant

**DOI:** 10.1007/s12471-026-02032-y

**Published:** 2026-02-23

**Authors:** Sietske A. Hogenhout, Jelmer R. Prins, Karin Y. van Spaendonck-Zwarts, Moniek G. P. J. Cox, Sanne J. Gordijn, Peter van der Meer, Carolien N. H. Abheiden

**Affiliations:** 1 Department of Obstetrics and Gynaecology, University of Groningen, University Medical Center Groningen, Groningen, The Netherlands; 2 Department of Cardiology, University of Groningen, University Medical Center Groningen, Groningen, The Netherlands; 3 Department of Genetics, University of Groningen, University Medical Center Groningen, Groningen, The Netherlands; 4 Department of Genetics, Leiden University Medical Center, Leiden, The Netherlands

## Introduction

Pregnant women with cardiomyopathy have an elevated risk for worsening heart failure, arrhythmias, and thromboembolic events [1]. Moreover, the offspring of these mothers with cardiomyopathy are at increased risk for adverse outcomes, including iatrogenic preterm birth, fetal growth restriction, and respiratory distress [2]. Therefore, the management strategy of these women in pregnancy requires careful monitoring and individualized therapeutic options to ensure the well-being of the mother and foetus. A specific genetic form of cardiomyopathy, caused by a variant in the phospholamban (PLN) gene, is predominantly seen in the Netherlands, the *PLN *p. (Arg14del) variant. Recently, preimplantation genetic testing (PGT) became available for *PLN* variant carriers, and the number of PGT requests for inherited cardiac diseases has increased rapidly [3]. Preconceptional and genetic counselling can help navigate within the complex interplay of potential cardiomyopathy-related health issues and pregnancy. However, the specific preferences of women with the *PLN* variant regarding these topics have not been studied yet.

## Methods

This prospective, observational study is conducted at the University Medical Center Groningen, the Netherlands. Women carrying the *PLN* variant were recruited in collaboration with the Dutch PLN Foundation. An online questionnaire was developed in collaboration with gynaecologists, cardiologists, a clinical geneticist, and representatives of the Dutch PLN foundation, covering preconceptional and genetic counselling preferences, pregnancy check-ups, and prior experiences with counselling. Participants were women carrying the *PLN* p.(Arg14del) variant, including those who had not been pregnant (yet). Age < 1 8 was an exclusion criterion.

## Results and discussion

In total, 40 women were included in this study. The mean age was 35.8 years (± 9.3), and 37.5% of the women were nulliparous. All women expressed a need for preconceptional counselling, preferably just before or during reproductive planning, together with their partners, and provided by a cardiologist and/or obstetrician. These findings differ from the study by Blomjous et al., who investigated the preferences for pre-pregnancy counselling in women with systemic lupus erythematosus (SLE) (*n* = 124), where only 5% preferred to receive information just before wishing to become pregnant [4]. These women with SLE preferred to receive information directly after diagnosis (53%), compared to only 20% of the women with the *PLN* variant, who prefer counselling directly after the diagnosis. This might be explained by the difference in age at diagnosis. Women with SLE are often diagnosed around childbearing age. In contrast, women with the *PLN* variant are often aware of their (possible) gene mutation at a younger age, due to the genetic nature. The information about potential effects on reproduction is less urgent at that time. As a result, they might want to wait until the counselling session is more relevant to them. 69% of the women with SLE preferred to receive the information together with their partner, which is in contrast to the 92.5% in our population. 77.5% of the women with the *PLN* variant prefers to receive the information from healthcare providers, consistent with the results of other studies on health information before, during and after pregnancy in women with SLE, rheumatoid arthritis and healthy women [4–6].

Most women (67.5%) with the *PLN* variant reported familiarity with invasive prenatal diagnostics (PND), such as chorionic villus sampling and amniocentesis, and 35.0% would consider it in a future pregnancy. Awareness and consideration of PGT was, 52.5% and 62.5%, respectively. The observed higher percentage of women considering PGT in future pregnancies compared to the percentage initially reporting familiarity with the technique may be explained by a lack of prior knowledge about PGT. After receiving information about PGT within the questionnaire, some participants may have developed a more favourable view of its use and thus expressed an intention to consider using it in future pregnancies. The fact that more women would consider PGT compared to PND is in line with earlier studies on several genetic disorders [7–9]. This can be explained by the fact that PGT offers the possibility to have a genetically unaffected child for the *PLN* variant, and termination of pregnancy can thus be avoided.

Among the women who have previously received preconceptional counselling, the majority appreciated this session and rated it as very good (33.3%) or good (41.7%), and more than 75% of them felt sufficiently informed to make their reproductive choices, which is consistent with findings in SLE and chronic kidney disease [4, 10].

## Conclusion

To conclude, women carrying the *PLN* p.(Arg14del) variant have a strong preference for preconceptional and genetic counselling, just before or at the time of reproductive intentions, together with their partners and preferably from the cardiologist or obstetrician. Additionally, a significant part of the women expressed to consider PGT in future pregnancies, a technique which is now available for this patient group in the Netherlands. These findings emphasize the importance of patient-centred counselling to support informed reproductive choices and improve patient satisfaction.
